# Laparoscopic Release for Median Arcuate Ligament Compression Syndrome Associated with a Celiac-Mesenteric Trunk

**DOI:** 10.1155/2022/3595603

**Published:** 2022-04-22

**Authors:** Shamir O. Cawich, Dave Harnanan, Lemuel Pran

**Affiliations:** Department of Surgery, University of the West Indies, St. Augustine Campus, Trinidad and Tobago West Indies

## Abstract

The median arcuate ligament compression syndrome is a rare entity that occurs in 2 per 100,000 unselected individuals. We present a case where the median arcuate ligament compression syndrome was associated with an equally uncommon anatomic variation—a celiac-mesenteric trunk, which occurs in 0.42-2.7% of unselected individuals. We could find no prior report of a celiac-mesenteric trunk being associated with the median arcuate ligament compression syndrome. This report also adds to the literature to show that a laparoscopic approach to median arcuate ligament release is feasible.

## 1. Introduction

Compression of the celiac trunk (CT) by the median arcuate ligament (MAL) may produce symptoms such as postprandial abdominal pain, weight loss, nausea, and vomiting [1]. This symptom complex is known by several names including Harjola-Marable syndrome, Dunbar syndrome, celiac artery compression syndrome, and median arcuate ligament syndrome [2].

The symptoms can become quite distressing and may require operative division of the MAL. Most authors recommend that this should be achieved using an open approach because it requires dissection of the aorta and its ventral branches, from which bleeding can be catastrophic. However, there are increasing numbers of reports, suggesting that the laparoscopic approach is becoming increasingly accepted for this procedure [2–4]. We report our experience with laparoscopic MAL release in a patient with a rare anatomic anomaly—the celiac-mesenteric trunk—thus adding to the existing literature to show that a minimally invasive approach is feasible even in the case of anatomic anomalies.

## 2. Report of a Case

A 44-year-old woman with no comorbidities presented to our facility with abdominal pain. There was no history of trauma, viral illnesses, or COVID vaccinations within the preceding 8 weeks. She complained of a six-month history of incapacitating epigastric pain, nausea, and vomiting that occurred within minutes of her meals. The symptoms were so severe that she developed anxiety before meals. In addition, she experienced unintentional loss of 25 pounds.

**Figure 1:**
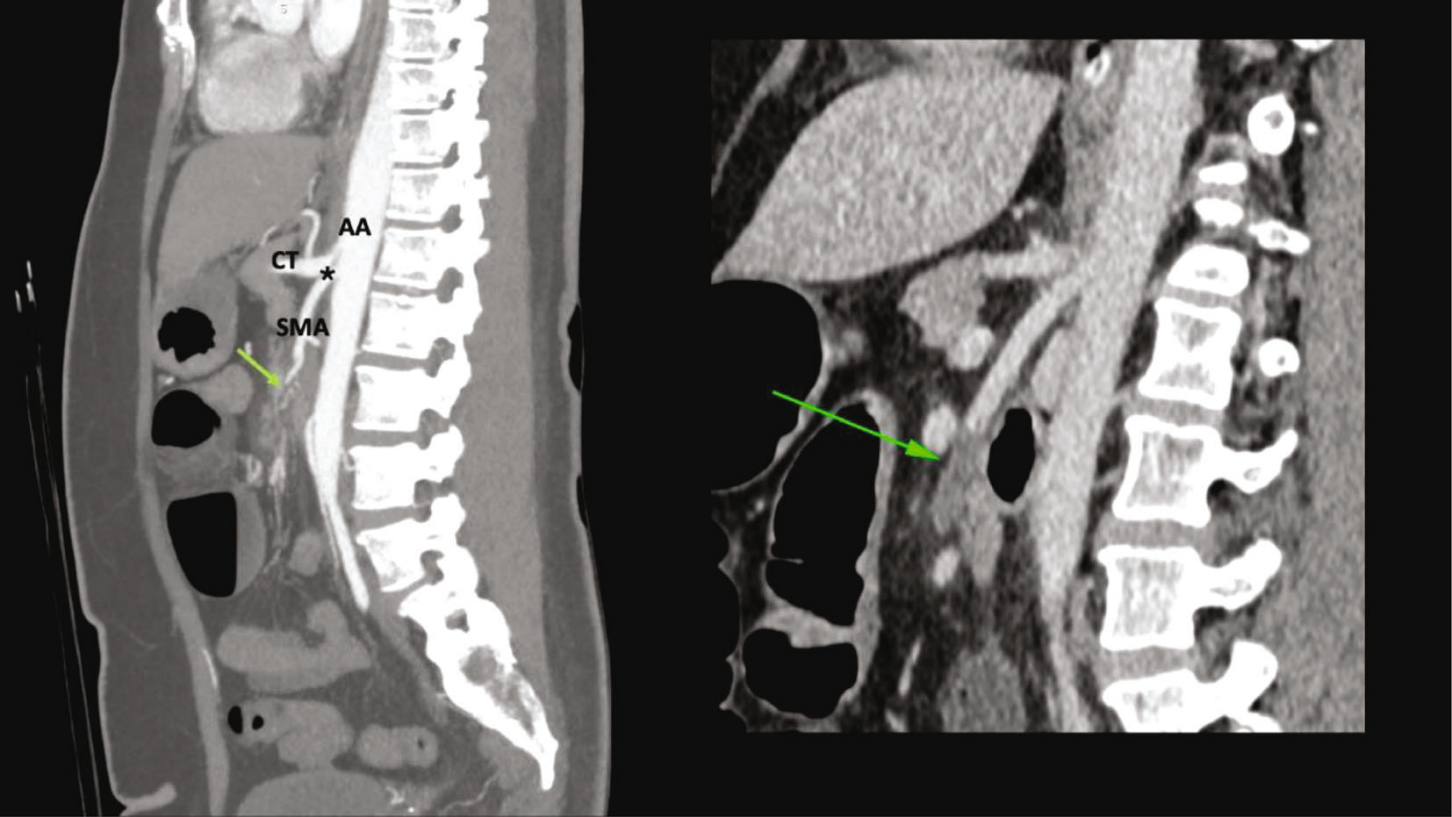
Sagittal view of a CT mesenteric angiogram showing a short celiac-mesenteric trunk (asterisk) arising from the abdominal aorta. The trunk further branches into the celiac trunk (CT) and superior mesenteric artery (SMA). Inset: an intraluminal thrombus can be seen within the SMA appearing as a filling defect (green arrow) and causing flow cessation in the lumen.

Persistent symptoms prompted computer tomographic mesenteric angiography. This revealed a variant celiac-mesenteric trunk (CMT) with evidence of celiac trunk/SMA compression and intraluminal thrombus in the SMA (Figure 1). Investigations did not reveal any other causes of thrombosis, including negative COVID swabs, normal blood coagulation studies, normal echocardiogram, and normal thoraco-aortograms.

**Figure 2:**
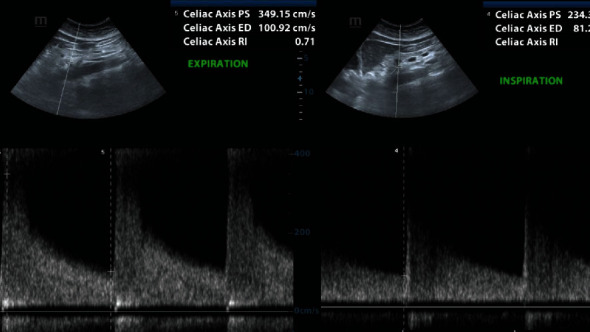
Duplex Doppler ultrasound images demonstrating narrowing of the CMT during forced expiration and dilation in the inspiratory phase. A maximum expiratory peak systolic velocity of 374 cm/sec was recorded within the coeliac axis.

Since this patient presented relatively late to the surgical team, it was thought that embolectomy would be difficult as we expected that the thrombus would be solidified. In addition, she had tolerated the luminal thrombus for six months without developing frank bowel necrosis. Therefore, a decision was made to commence therapeutic anticoagulation. Despite anticoagulation for four weeks, her symptoms persisted and prompted repeat angiography at six weeks. At this point, the thrombus was no longer present, but there was now perijejunal fat stranding, oral contrast hold-up, and mural thickening, suggestive of an ischemic jejunal stricture. A Doppler ultrasound was performed, and this revealed cyclic angulation and compression of the celiac trunk on forced expiration, with expiratory dilatation (Figure 2). A maximum expiratory peak systolic velocity of 374 cm/sec was recorded within the celiac axis and secured our diagnosis of MAL syndrome.

**Figure 3:**
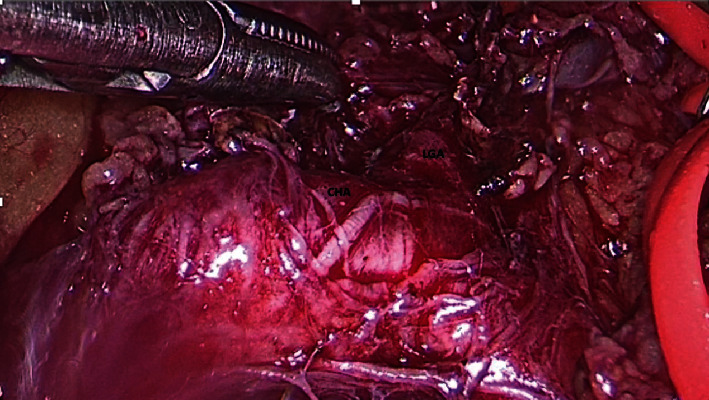
Intraoperative view showing that the left gastric artery (LGA) and common hepatic artery (CHA) have been dissected. The vessel will be encircled with loops and followed retrograde to the celiaco-mesenteric trunk.

This patient was offered a laparoscopic release of the MAL with resection of the jejunal stricture and primary anastomosis. The patient was prepared for anaesthesia, and a 12 mmHg pneumoperitoneum was created. The operation commenced by interrupting the hepato-gastric ligament to enter the lesser sac. The left gastric artery was identified and traced retrograde to the CMT trunk (Figure 3).

**Figure 4:**
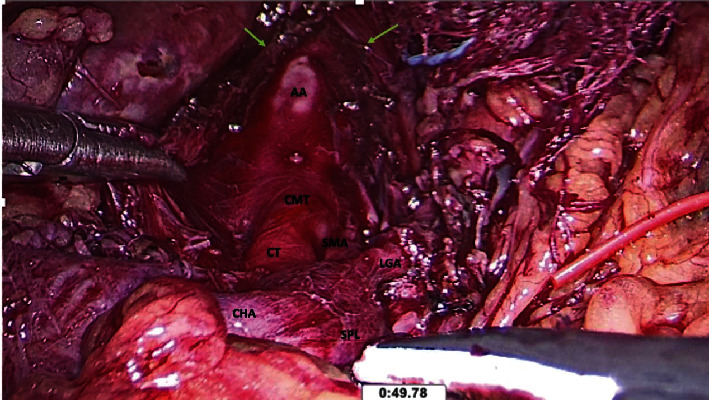
Completed dissection reveals a 5 cm segment of the ventral abdominal aorta (AA). The short celiaco-mesenteric trunk (CMT) can be seen branching into the superior mesenteric artery (SMA) and celiac trunk (CT). The three branches of the celiac trunk can be traced: common hepatic artery (CHA), splenic artery (SPL), and left gastric artery (LGA). The fibers of the divided median arcuate ligament are also visible (arrows).

We carefully and patiently dissected at the superior aspect of the CMT using blunt instruments in order to avoid arterial injury. For this, we used combinations of a peanut gauze swab and a 5 mm LigaSure™ Maryland dissector (Medtronic, Minneapolis, USA), taking care to remain in the avascular adventitial plane. This allowed us to expose the ventral part of the aorta. Once the CMT and the aorta were clearly identified and separated from the MAL, the release was performed using the LigaSure TM (Medtronic, Minneapolis, USA). An immediate and audible release resulted from MAL division. The MAL was divided until the ventral surface of the aorta was exposed for 5-6 cm (Figure 4).

**Figure 5:**
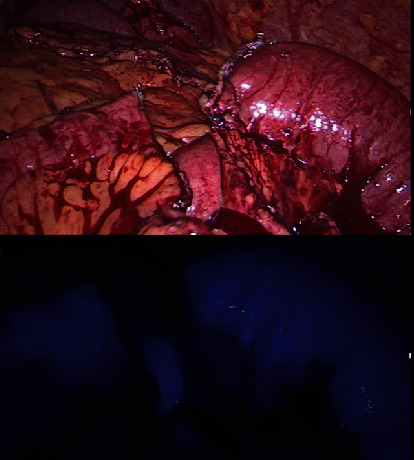
Intraoperative view of the laparoscopic feed. The strictured jejunal segment has been resected using an endo-GIA stapler (a). In order to confirm perfusion of the jejunal ends, ICG has been injected intravenously (b), and perfusion was confirmed.

The jejunal stricture was then identified and resected, with primary intracorporeal anastomosis. In order to confirm viability and perfusion of the anastomotic ends, we used fluorescence angiography with intravenous indocyanine green (Figure 5). Once mesenteric flow and viability of the resected bowel ends were confirmed, a primary stapled anastomosis was completed and the specimen removed. The operation was completed in 120 minutes without a recorded complication.

**Figure 6:**
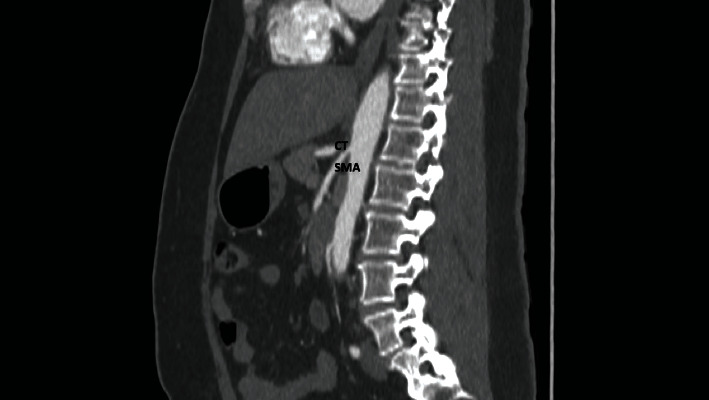
Follow-up CT scan at 8 months showing resolution of the thrombus in the superior mesenteric artery.

Eight months after her operation, there has been complete resolution of symptoms and thirty-one pounds of weight gain. A follow-up CT scan revealed complete resolution of the thrombus (Figure 6) and resolution of intra-abdominal findings, such as fat stranding and mural thickening.

## 3. Discussion

The patient in this report had a CMT—an anatomic variation first described by Lipshutz [5]. He coined the term “*truncus celiaco-mesenterica*” to describe 2 cadavers with a variant where the SMA and CT took a common origin from the aorta. Published data suggest that the CMT occurs in 0.42% [6] to 2.7% [7] of unselected persons across the globe. In the Caribbean population, it occurs in 0.45% of autopsies [8] and is commoner in males. According to the classification proposed by Wang et al. [9], this was a short trunk (<15 mm from aortic origin), type I variant (all 3 coeliac trunk branches having a common origin with superior mesenteric artery). The anatomic variant detected here is uncommon and has only been reported in association with the MAL syndrome in one prior report by Chaiwatcharayut et al. [10].

In traditional anatomic descriptions, the aorta passes behind the MAL of the diaphragm into the abdomen at the twelfth thoracic vertebral level [5]. Approximately 1 cm distal to the MAL, the celiac trunk arises as the first ventral branch of the abdominal aorta [5]. However, in approximately 10-20% of persons, the MAL is located caudally and compresses the celiac artery and adjacent sympathetic ganglia [5, 11, 12]. The clinical syndrome was first described in 1963 by Harjola, a Finnish surgeon who also described the first recorded MAL release [11]. Later in 1965, Dunbar et al. [12], radiologists, published a series of 15 cases. Both names have been eponymously attached to this syndrome.

The MAL syndrome is also a rare phenomenon in which the celiac trunk is compressed as it passes behind the diaphragm [1]. It is reported to occur in 2 per 100,000 persons and is approximately four times more common in women, especially those between the ages of 30 and 50 years of age [13]. While most persons with a CMT are asymptomatic [7, 8], we can only surmise that the presence of a CMT may have predisposed this patient to the MAL syndrome. There has only been one prior report of this association between the CMT and MAL syndrome described previously in the medical literature [10].

There is controversy about the pathophysiology producing this syndrome. One theory is that compression becomes more marked as the diaphragm is depressed during inspiration, producing ischaemia in the organs supplied by branches of the celiac trunk [1]. An alternative theory is that compression of the sympathetic ganglion in the celiac trunk produces neuropathic pain [1, 13] and hyperstimulation of the celiac ganglion produces splanchnic vasoconstriction [1, 13]. The prevailing theory is that both mechanisms may be involved to produce the characteristic triad of vomiting, postprandial pain, and weight loss [1, 3].

Because the symptoms are nonspecific and pathophysiology is poorly understood, there has been no consensus on the criteria for diagnosis. Therefore, the diagnosis is largely made by excluding alternative diagnosis that may explain the patient's symptoms [2, 3, 14]. Often, a Doppler ultrasound is the initial diagnostic test. This may demonstrate the stenotic vessel and dynamic flow variations [3]. It is a readily available, inexpensive investigation that avoids exposure to radiation [3, 14].

The point of compression can be identified by digital subtraction angiography, computer tomographic angiography, or magnetic resonance angiography [4, 15]. CT/MR offers the advantage of being noninvasive, able to identify concurrent intra-abdominal pathology and exclude differential diagnoses. In our case, CT angiography and DSA were performed and supported the diagnosis. The features on angiography that most authors consider diagnostic are (1) demonstrating the area of compression, (2) cephalad movement of the celiac axis during inspiration, and (3) poststenotic dilation upon expiration [16, 17]. All three features were present in our case.

Once the diagnosis is made, operative decompression is required. This involves division of the MAL and dissection of periaortic tissues to interrupt neural tissue that may be responsible for neuropathic pain [14]. This operation was traditionally performed using the open approach because the dissection required exposure of the abdominal aorta and the celiac trunk, with the potential for catastrophic bleeding in the event of an injury. However, in the past decade, there have been increasing reports of laparoscopic celiac decompression [14].

The benefits of a laparoscopic approach are similar to those for other major open operations and include reduced postoperative pain, wound complications, respiratory sequelae, gastrointestinal disfunction, and hospitalization [1]. We acknowledge the concern that catastrophic bleeding may occur if there is iatrogenic injury to the aorta during dissection. However, we found that the periarterial adventitial plane provided an avascular plane for dissection. Therefore, we advocate patient dissection with blunt instruments and care to stay in the adventitial plane. We found the use of a laparoscopic peanut gauze and the LigaSure™ Maryland dissector useful to expose the correct dissection plane.

Operative decompression of the MAL is considered to be the gold standard treatment. The largest contemporary series reported that 75% of patients remained symptom-free after MAL release with mean follow-up of nine years [17]. Endovascular techniques such as angioplasty and stenting have been attempted, but they have been largely ineffective as a therapeutic maneuver as they do not effectively address the extrinsic compression of the celiac trunk [3, 14, 16].

In order to confirm complete decompression, intraoperative Doppler assessment and/or angiography may be considered. In our case, we used fluorescence angiography with intravenous indocyanine green to confirm mesenteric perfusion and small bowel viability. Fluorescence angiography with intravenous indocyanine green is a relatively new technique that allows the surgeon to evaluate perfusion in transected viscera in real time. Recently, it has gained application in laparoscopic colorectal and foregut surgery as well as bariatric procedures to reduce the incidence of anastomotic leaks. We have not encountered reports of this application in patients with MAL syndrome and suggest that it may be useful as an alternative to intraoperative Doppler evaluation. In the event of persistent stenosis after decompression, the other therapeutic options are aorto-celiac bypass or celiac artery angioplasty [1].

## 4. Conclusion

Although there are increasing reports of the MAL syndrome in medical literature, this case was unique because it is the second recorded case in which there has been an association between the presence of a celiac-mesenteric trunk, an uncommon anatomic anomaly. It is likely that the anatomic anomaly in this patient contributed to the symptomatology from the MAL syndrome.

In addition to noting this association, we also add to the literature by suggesting that fluorescence angiography with intravenous indocyanine green may be used to confirm mesenteric perfusion following operative decompression. Finally, we also add to the growing body of literature that a laparoscopic approach is feasible for MAL decompression.

## Data Availability

All pertinent data will be available upon request from the chief author.
